# Combinatorial DNA Rearrangement Facilitates the Origin of New Genes in Ciliates

**DOI:** 10.1093/gbe/evv172

**Published:** 2015-09-02

**Authors:** Xiao Chen, Seolkyoung Jung, Leslie Y. Beh, Sean R. Eddy, Laura F. Landweber

**Affiliations:** ^1^Department of Molecular Biology, Princeton University; ^2^Janelia Research Campus, Howard Hughes Medical Institute, Ashburn, Virginia; ^3^Department of Ecology and Evolutionary Biology, Princeton University; ^4^Present address: Howard Hughes Medical Institute, Department of Molecular & Cellular Biology, and John A. Paulson School of Engineering and Applied Sciences, Harvard University

**Keywords:** novel genes, gene duplication, alternative splicing, genome rearrangement, comparative genomics

## Abstract

Programmed genome rearrangements in the unicellular eukaryote *Oxytricha trifallax* produce a transcriptionally active somatic nucleus from a copy of its germline nucleus during development. This process eliminates noncoding sequences that interrupt coding regions in the germline genome, and joins over 225,000 remaining DNA segments, some of which require inversion or complex permutation to build functional genes. This dynamic genomic organization permits some single DNA segments in the germline to contribute to multiple, distinct somatic genes via alternative processing. Like alternative mRNA splicing, the combinatorial assembly of DNA segments contributes to genetic variation and facilitates the evolution of new genes. In this study, we use comparative genomic analysis to demonstrate that the emergence of alternative DNA splicing is associated with the origin of new genes. Short duplications give rise to alternative gene segments that are spliced to the shared gene segments. Alternative gene segments evolve faster than shared, constitutive segments. Genes with shared segments frequently have different expression profiles, permitting functional divergence. This study reports alternative DNA splicing as a mechanism of new gene origination, illustrating how the process of programmed genome rearrangement gives rise to evolutionary innovation.

## Introduction

New gene origination is an essential feature of genome evolution. New genes arise mainly through gene duplication, retrotransposition, lateral gene transfer, exon shuffling, and de novo origination from noncoding sequences (Long et al. 2003, 2013; Kaessmann 2010). Among these mechanisms, exon shuffling and alternative exon splicing permit the modular assembly of new genes, and both greatly enhance eukaryotic protein diversity and may contribute to the evolution of many novel biological functions (Gilbert 1978; Patthy 1999, 2003; Liu and Grigoriev 2004; Keren et al. 2010).

**Fig. 1.—:**
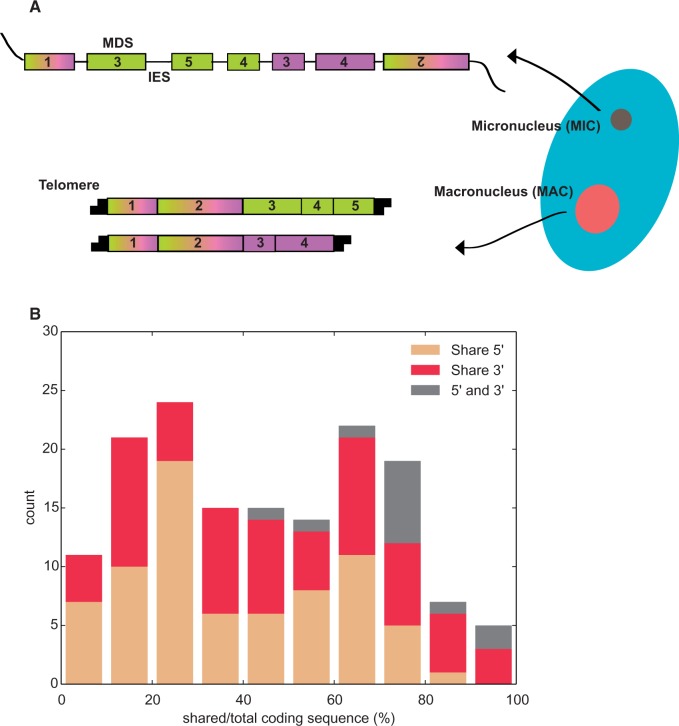
Alternative DNA processing in *Oxytricha trifallax*. (*A*) After sexual reproduction, a new MAC develops from a copy of the MIC. In the MIC, MDSs are interrupted by IESs and can be disordered or inverted. During development, IESs are deleted and MDSs are stitched together, some requiring inversion or unscrambling, followed by chromosome fragmentation and telomere addition. Some MDSs (e.g., shared MDSs 1 and 2, shown in purple-green blend) are processed to contribute to more than one MAC chromosome. Alternative MDSs are marked in purple and green, respectively. (*B*) Distribution of the ratio between the lengths of shared coding regions and the lengths of total coding regions in cases of genes that share 5′ or 3′ regions, or both. In cases where an MDS is shared by multiple genes, the shortest shared portions are shown.

Ciliates are unicellular eukaryotes that undergo extensive genome rearrangements during development of a specialized somatic nucleus from archival germline nucleus (Prescott 1994). In *Oxytricha trifallax*, the somatic macronucleus (MAC) consists of over 16,000 short “nanochromosomes” that average just 3.2 kb and encode just 1–8 genes (Swart et al. 2013), while the germline micronucleus (MIC) exhibits a complex genome architecture, comprising ∼225,000 short genic segments (macronuclear destined sequences, MDSs) interrupted by brief noncoding sequences (internal eliminated sequences, IESs) (fig. 1*A*). Furthermore, the MDSs retained in the soma are often present in the germline in a permuted order or inverse orientation. These are referred to as scrambled MDSs. Therefore, correct assembly of *Oxytricha*’s set of ∼18,000 functional somatic genes requires precise deletion of IESs and extensive reordering and inversion of tens of thousands of DNA segments. The recent sequencing of the *O. trifallax* MAC (Swart et al. 2013) and MIC genomes (Chen et al. 2014) revealed over a hundred cases of alternative MDS processing (Chen et al. 2014), where a single MDS in the MIC can contribute to multiple distinct somatic genes (fig. 1*A*). Similar to alternative splicing and exon shuffling––but at the DNA level, this phenomenon that David Prescott called “MDS shuffling” could contribute to the creation of novel genes (Prescott 1999; Katz and Kovner 2010; Chen et al. 2014).

Some previous studies used RNA-Seq data to infer possible cases of alternative MDS processing in the ciliate *Chilodonella uncinata *(Gao et al. 2014, 2015) but most cases have not been confirmed by complete germline and somatic DNA sequences. Zhou et al. (2011) reported a case of MDS duplication and shuffling that produced a novel gene in *Oxytricha*, but without alternative DNA assembly. The *Oxytricha* germline genome project (Chen et al. 2014) reported 105 cases of alternative MDS processing that are unambiguously supported by comparison of germline and somatic genome sequences. Here, we analyze these cases of alternative DNA splicing from an evolutionary perspective. We sequenced and compared genomes for related ciliate species with similar genome architectures to investigate the origin and evolution of both alternative MDS processing and the de novo genes that this process has generated.

## Materials and Methods

### Filtering of Alternative MDS Processing Cases

*Oxytricha* chromosomes that share MDSs were filtered to remove the following cases:

1. Noncoding chromosomes, that is, those lacking gene predictions.
2. Multigene chromosomes that share exactly one gene, with 100% overlap and sequence similarity in the coding sequence of the shared gene.
3. Chromosomes that only share noncoding regions. The shared region either does not extend into coding sequences or extends less than 20 nt into coding sequences.

### Genome Sequencing and Assembly

In this study, we sequenced and assembled the macronuclear genomes of six stichotrich ciliates *Urostyla* sp., *Paraurostyla* sp., *Laurentiella* sp., *Stylonychia lemnae*, *Tetmemena* sp., and *Sterkiella histriomuscorum*, as well as *O. trifallax* strain JRB510.

The stichotrich DNA came from various sources, including Chang et al. (2005). *Urostyla* sp. (probably *U. grandis*) DNA was a generous gift from the late David Prescott (University of Colorado, Boulder). *Paraurostyla* sp. (probably *P. weissei*) was originally isolated by Mann Kyoon Shin (University of Ulsan, Korea) from lakes and soils in the Princeton, NJ, area. *Laurentiella* sp. was collected by Tom Doak (Indiana University) from a puddle on Princeton University campus. *Stylonychia lemnae* strain 2 × 8/2 was the same strain described in Jung et al. (2011). *Sterkiella histriomuscorum* strain BA was the same strain described in Zoller et al. (2012). DNA was extracted as previously described in Chang et al. (2005). Illumina libraries with an insert size of 300 bp were prepared and sequenced in paired-end mode (2 × 100 bp reads) on a HiSeq 2500 machine, producing ∼35 million read pairs for each genome.

The *O. trifallax* strain JRB510 was cultured in Pringsheim salts (0.85 mM Ca(NO_3_)_2_, 0.11 mM Na_2_HPO_4_, 0.35 mM KCl, 0.08 mM MgSO_4_, pH 7.0) at room temperature, with *Chlamydomonas reinhardtii* as a food source. *Oxytricha* cells were starved for ∼ 16 h, and then harvested for macronuclear isolation experiments as previously described in Lauth et al. (1976). Cells were lysed through ten strokes of a Kontes pestle B dounce homogenizer on ice. Genomic DNA was subsequently purified from macronuclei using a Nucleospin Tissue Kit (Macherey-Nagel) and subject to single-read sequencing on an Illumina HiSeq 2500, according to manufacturer’s instructions, producing 90 million, 170-bp long reads. *Tetmemena* sp. was originally found growing together with *Oxytricha nova* and then cultured individually. It is similar to *Tetmemena pustulata*, formerly *Stylonychia pustulata*. Cells were cultured and DNA was collected following the same procedures as for *Oxytricha* JRB510. The DNA was prepared into Illumina libraries with an insert size of 400 bp and sequenced in paired-end mode (2 × 215 bp reads).

For genome assembly, SPAdes (3.1.0) (Bankevich et al. 2012) was run with the BayesHammer error correction algorithm (Nikolenko et al. 2013) and the “careful” option. Trinity (v20140413) (Grabherr et al. 2011) was run with default parameters on the error-corrected reads output by SPAdes. CAP3 (Huang and Madan 1999) was used to merge the two assemblies (parameters: -o 40 –p 98). Telomeric reads were aligned to the assembly using BLAT (default parameters) (Kent 2002) and contigs missing one or two telomeres were extended and “capped” using custom Python scripts if telomeric reads mapped to their ends. Contigs with a GC content above 0.45 were removed to filter out bacterial contamination. The program CD-HIT (Fu et al. 2012) was run to cluster sequences at 95% sequence similarity (parameters: -c 0.95 -aS 0.9 -uS 0.1).

For the *Oxytricha* JRB510 genome, the CAP3-merged and telomere-extended assembly was aligned to the *Oxytricha* JRB310 MAC genome (Swart et al. 2013) using BLASTN (BLAST+, default parameters) (Camacho et al. 2009). If a JRB310 contig is >90% covered with >90% sequence similarity by a JRB510 contig, the latter was selected to be included in the final assembly and renamed according to its JRB310 ortholog. The remaining contigs were first filtered by removing sequences with a GC content above 0.45 (to filter out bacterial contigs). Then contigs that did not contain any telomere and any match longer than 200 bp to the JRB310 genome were eliminated. “Chaff” contigs under 500 bp were removed if they matched contigs longer than 500 bp with >80% coverage and >90% sequence similarity. The program CD-HIT (Fu et al. 2012) was run to cluster sequences at 95% sequence similarity (parameters: -c 0.95 -aS 0.9 -uS 0.1).

The length statistics for our genome assemblies are shown in table 2. The majority of MAC chromosomes were assembled completely as two telomere contigs, which facilitated gene prediction and the identification and analysis of orthologs.

### Gene Prediction, Ortholog Identification, and Functional Annotation

Gene prediction was performed with Augustus (version 2.5.5) (Stanke and Morgenstern 2005) using the *Oxytricha* model (Swart et al. 2013) for all genomes except *Euplotes*. Predicted protein sequences were aligned across all species with BLASTP (BLAST+, parameters: -query_gencode 6 -*e*-value 1e-7; Camacho et al. 2009) and OrthoMCL (Li et al. 2003) was used to assign orthologous groups (default parameters). For *Euplotes*, we ran BLASTX (BLAST+, parameters: -query_gencode 10 -e-value 1e-7; Camacho et al. 2009) to search for orthologs. Protein domains were determined using HMMER version 3.0 (Finn et al. 2011) search with default parameters against the Pfam-A profile HMM database (version 26.0) (Punta et al. 2012). The output was filtered using independent *e*-value ≤ 0.001 and conditional *e*-value ≤ 0.1 for at least one domain match in potentially repeated domains. InterProScan version 5.5 (Jones et al. 2014) was also used to annotate protein domains. We compared the protein domains in alternative MDS regions in each group of genes that share MDSs. The presence of different domains in the alternative MDS regions that are spliced to the same shared MDSs would suggest novel protein domain organizations.

We used RNA-seq data reported previously by Swart et al. (2013) to quantify gene expression patterns. Expression profiles were created by mapping RNA-seq reads to alternative MDS regions with BLAT (Kent 2002) (default parameters). The BLAT output was filtered for >90% alignment and >94% identity. RNA-seq counts were normalized with DESeq (Anders and Huber 2010) using the default method, as described in Swart et al. (2013). DNA copy number was assessed by mapping genomic DNA sequencing reads (Swart et al. 2013) to alternative MDS regions with BLAT (Kent 2002) (default parameters) and filtering for >90% alignment and >94% identity.

### Assessment of Genome Assembly Completeness

We assessed the completeness of our genome assemblies using two methods: The number of conserved core eukaryotic genes (CEGs) and the number of tRNA genes. For the CEG analysis, we searched for homologs of 248 CEGs (Parra et al. 2007) using BLASTP (Camacho et al. 2009) and filtered out matches with *e*-values higher than 1e-6 or <70% coverage of the CEG sequence. Most of CEGs missing BLASTP matches could be found using HMMER3 domain searches as described in Swart et al. (2013). We queried the superset of 245 CEGs from *Oxytricha*, *Paramecium*, and *Tetrahymena*. All assemblies contain homologs of all 245 CEGs except the MAD2 spindle assembly checkpoint protein (KOG3285), which is also missing from *Oxytricha*. Therefore, our stichotrich assemblies are complete. We also predicted tRNAs using tRNAscan-SE (version 1.3.1, default parameters) (Lowe and Eddy 1997). All assemblies contain a complete set of tRNAs encoding for the 20 standard amino acids, as well as selenocysteine.

### Construction of Phylogenetic Trees

The phylogeny of the ciliate species in this study was inferred using PhyML (with the HKY85 substitution model and 100 bootstrap replicates; Guindon et al. 2010) based on a concatenated multiple sequence alignment of 18S and 28S ribosomal RNA (rRNA) genes with MAFFT (v6.956b) (Katoh et al. 2002) using the default parameters. We also produced phylogenetic trees for paralogous proteins with shared MDSs to determine the timing of duplication. Alternative (unique) MDS regions were aligned with MAFFT and excess gaps and poorly aligned regions were removed with trimAl (version 1.2, with the “-automated1” parameter) (Capella-Gutiérrez et al. 2009). Phylogenetic trees were generated from the alignments using PhyML with a single substitution rate category and the Jones, Taylor, and Thorton (JTT) substitution model, optimized for tree topology and branch length (parameters: -d aa -b 100 -m JTT -o tl -c 1), as well as MrBayes v3.2.2 (Huelsenbeck and Ronquist 2001) (parameters: prset aamodelpr = mixed; mcmc nchains = 1 ngen = 300,000). Trees were drawn using FigTree 1.4.2 (http://tree.bio.ed.ac.uk/software/figtree/, last accessed September 27, 2015).

### Evolutionary Rate Analysis

For each gene we extracted coding and protein sequences from both alternative and constitutive MDS regions. Amino acid substitution rates were calculated from pairwise protein alignments (MAFFT version 6.956b; Katoh et al. 2002) between JRB310 and JRB510 orthologs. Protein alignments were converted to coding sequence alignments using PAL2NAL (Suyama et al. 2006). Nonsynonymous to synonymous rate (d*N*/d*S*) ratios were calculated using the codeml program in PAML (Yang 2007) (version 4.5) with parameters “icode = 5, runmode = −2, CodonFreq = 2”. For this analysis, we included only cases where both alternative and constitutive coding regions are longer than 150 nt and d*S* values are between 0.01 and 5.

## Results

We analyzed 105 cases of alternative DNA processing identified in the *O. trifallax* micronuclear genome (Chen et al. 2014). We excluded cases that do not involve new genes, including noncoding chromosomes, multigene chromosomes that share exactly one gene, and chromosomes that only share noncoding regions (see Methods). The remaining 69 cases involve 69 germline loci that encode MDS segments for 153 MAC chromosomes with shared 5′ or 3′ terminal regions, or both (table 1). Figure 1*B* shows the distribution of the fraction of shared coding regions relative to the total length of the coding region. This portion ranges from just a few percent to over 90%. There is no strong bias for sharing of 5′ versus 3′ end regions. Most loci contain two genes that share single-copy MDSs. There are six loci that each contain a set of three genes with shared MDSs. Three loci contain four genes that share MDSs, and there exists one locus that gives rise to five such genes.

**Table 1:** Summary of Alternative MDS Processing Cases Investigated in this Study

	Total	Share 5′	Share 3′	Share 5′ and 3′	No. of Nonscrambled	No. of Scrambled
No. of MIC loci	69	32	31	6	31	38
No. of MAC chromosomes	153	75	65	13	80	73
No. of MDSs	2,420	330	349	183	2,191	229

### Alternative MDS Processing Creates New Genes

We investigated the emergence of these 69 cases of alternative DNA splicing by examining their orthologs in related species. We sequenced and assembled the macronuclear genomes of six stichotrich ciliates *Urostyla* sp., *Paraurostyla* sp., *Laurentiella* sp., *Stylonychia lemnae*, *Sterkiella histriomuscorum*, and *Tetmemena* sp., whose ribosomal DNA has a closest hit (98%) to *Tetmemena pustulata* ribosomal DNA (GenBank accession AF508775). We also used the preliminary macronuclear genome assembly of *Euplotes crassus*, an earlier diverging Spirotrich ciliate, described in Swart et al. (2013). The assembled stichotrich genomes contain a large percentage of completely assembled somatic chromosomes (table 2). Analyses of CEGs and tRNA complement suggest that our assemblies are complete (see Methods). Aeschlimann et al. (2014) previously reported a *Stylonychia lemnae* macronuclear genome assembly for a different strain 130c, whose assembly size (50.2 Mb) and total number of contigs (19,851) and two telomere contigs (16,059) are similar to our *Stylonychia* assembly.

**Table 2:** Length Statistics of Stichotrich Genome Assemblies

	N20	N50	Average Length	Total Complexity	No. of Contigs	5′ Telomere	3′ Telomere	Both Telomeres
*Urostyla* sp.	5,571	2,898	2,105	42.62M	20,244	15,569	15,960	13,496
*Sterkiella histriomuscorum*	5,058	2,822	2,011	66.36M	32,996	19,368	20,756	16,924
*Stylonychia lemnae*	5,643	3,089	2,333	54.71M	23,449	19,324	19,443	18,058
*Laurentiella* sp.	5,466	3,043	2,293	49.04M	21,383	17,789	17,766	16,399
*Paraurostyla* sp.	5,326	2,882	2,249	57.10M	25,391	21,028	21,019	19,135
*Tetmemena* sp.	5,714	3,312	2,404	60.63M	25,219	18,718	18,166	16,577
*Oxytricha trifallax* JRB510	5,767	3,392	2,558	57.45M	22,458	18,055	18,335	15,918

**Fig. 2.—:**
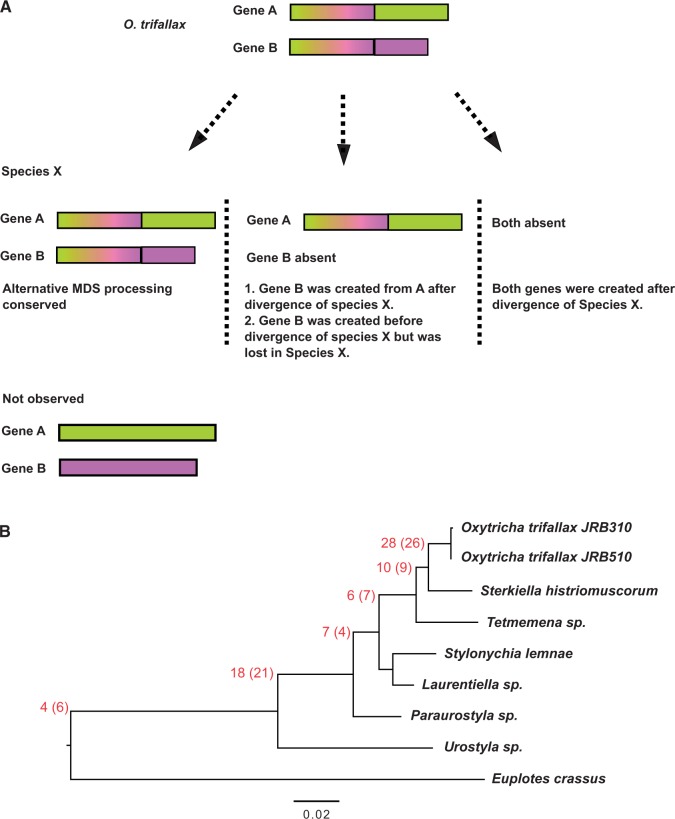
The presence of alternative DNA processing is associated with the emergence of new genes. (*A*) Inference of the origin of alternative MDS processing based on the presence of orthologs and MDS sharing in other ciliates. (*B*) Mapping of all cases of alternative MDS processing onto a phylogeny generated from 100 bootstrap replicates with PhyML (with the HKY85 substitution model) based on a MAFFT concatenated multiple sequence alignment of 18S and 28S rRNA genes from 8 ciliate species, including 2 *Oxytricha trifallax* strains. The tree is rooted with *Euplotes crassus*. All bootstrap values are above 90%. The scale below the phylogeny illustrates branch substitutions per site. Numbers in red at the tree nodes represent the inferred numbers of cases of alternative processing that emerged before the divergence at each node. The numbers in parenthesis indicate corrected values after examining individual phylogenetic trees, which reveal the loss of paralogs in a few cases.

For two genes A and B that share MDSs in *Oxytricha*, we queried the presence of their orthologs in other species and assessed whether their orthologs also share sequences, which would suggest that they are also products of alternative MDS processing. Our query in any species X yielded three possible scenarios (fig. 2*A*). First, the presence of both orthologs that shared sequences could suggest the conservation of alternative DNA processing. Second, the presence of only the ortholog of A would suggest the creation of novel gene B via the reuse of a subset of existing segments for gene A after the divergence of species X. Another possibility in this case is that B was created before the divergence of species X but later lost from species X. Third, the absence of either ortholog would suggest that both genes were created after the divergence of species X. If no other species contains either ortholog, this would suggest that both genes were new to the *Oxytricha* lineage and that an intermediate species with just one gene should exist but was not included in our survey.

Because we never observed a case where both orthologs are present but they do not share MDSs, we conclude that the emergence of alternative MDS processing is associated with the creation of new genes (gene B) from an existing gene (gene A), by reuse of some of gene A’s germline precursor segments. We mapped the number of new genes created in each lineage onto a phylogeny (fig. 2*B*). All examples appear to have originated in the stichotrich lineages (i.e., none are conserved in *Euplotes*) and a large number (28) appear specific to the *Oxytricha* lineage and thus probably emerged fairly recently. This is a parsimonious estimate, given the possibility that some genes could have emerged earlier but been lost in some species. Corrections are discussed in the next section. In cases where two orthologs share MDSs, the length of the shared regions is usually conserved relative to that in *Oxytricha* (all but 4 are similar within 50 bp or 10%, whichever is larger, of the length of the shared gene segments in *Oxytricha*).

### Most Alternative MDSs Derived from Segmental Duplications

**Fig. 3.—:**
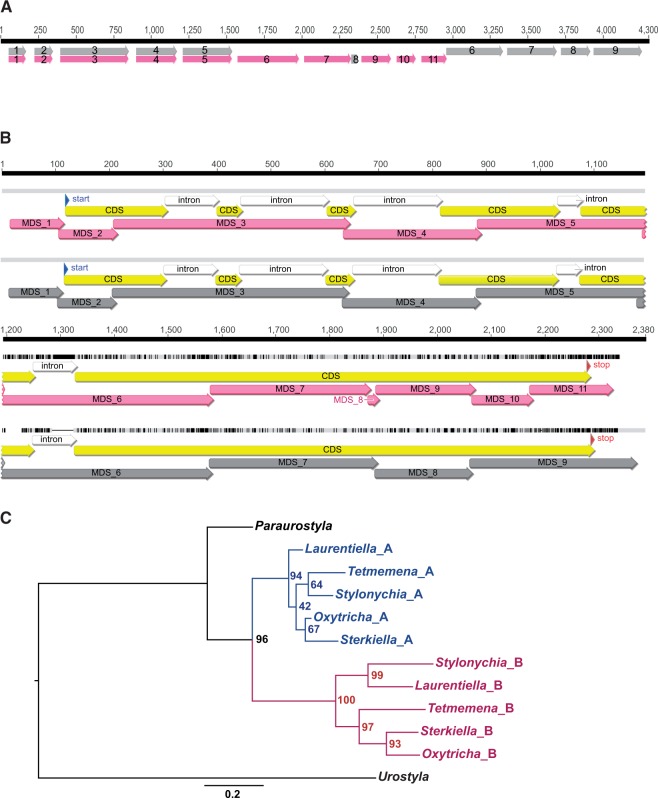
Duplication is the major mechanism for creation of alternative gene segments. (*A*) Germline map of a locus with two nonscrambled genes that share five DNA segments at the 5′ end. Arrows represent MDSs and gaps represent IESs. Gray: Contig8.0; pink: Contig22835.0. (*B*) Translated alignment (nucleotide alignment guided by amino acid sequence) of the MAC contigs from Panel A showing paralogy between the duplicated MDSs downstream of MDS 5 (MDS 1–5 are shared) and that the locations of MDS boundaries are conserved between the two paralogs (conserved precisely between MDS 6 and 7 in both pink and gray; 1 bp different in location between MDS 8 and 9 in pink and MDS 7 and 8 in gray; 3 bp different between MDS 9 and 10 in pink and MDS 8 and 9 in gray). Unique bases or gaps on each sequence are annotated with a vertical black bar, and identical regions are highlighted in light gray. Wide arrows in different colors represent exons (labeled as CDS, yellow), introns (white), start and stop codons, and MDSs. The overlaps between MDSs contain short regions of microhomology at recombination junctions. (*C*) A maximum-likelihood tree, constructed using the alternative MDS regions of two paralogous genes that share MDSs, shows accelerated evolution of gene B after MDS duplication. Gene A: Contig13046.0; gene B: Contig12964.0. The phylogeny, rooted with the *Urostyla* ortholog, was generated by PhyML with a single substitution rate category and the JTT substitution model, optimized for tree topology and branch length. Numbers at the tree nodes indicate bootstrap values for 100 replicates. The multiple sequence alignment was produced with MAFFT v6.956b (default parameters) and trimmed with trimAl v1.2 with the “-automated1” parameter to remove excess gaps and poorly aligned regions. The scale below the phylogeny illustrates branch substitutions per site.

For each group of *Oxytricha* genes that share MDSs with each other, we compared the unique alternative MDS sequences, that is, the regions (often the 5′ or 3′ ends) that differ from each other in the mature chromosomes, with each other. The majority of these (54 out of 69 cases) are more than 40% similar at the protein level (BLASTP, alignment length >80% of the unique regions and *e*-value <1e-10; Camacho et al. 2009), suggesting that the new, alternative segments arose by duplication of ancestral MDSs. Duplication of partial gene loci most likely occurred, instead of entire genes. It is also possible that duplication of complete gene loci was followed by partial loss of gene regions, resulting in the requirement for sharing of the missing segments (similar to a proposed model for the origin of scrambled genes; Gao et al. 2015), although careful examination of neighboring MIC sequences did not reveal traces of degenerate or lost duplicate copies of the constitutive MDSs. Figure 3*A* shows the germline MDS–IES map for two paralogous genes with shared MDSs. Their germline precursor loci overlap, with the alternatively spliced MDSs downstream of the shared, constitutive MDSs. Figure 3*B* shows a translated alignment of the somatic versions of both sequences. The boundaries between segments 6 and 7 in the duplicated, alternative regions are precisely conserved in location between the two genes, including short regions of microhomology at recombination junctions (marked by the overlap between consecutive MDSs). The boundaries between segments 8 and 9 in the pink gene and segments 7 and 8 in the gray gene differ in location by just 1 bp, and the boundaries between segments 9 and 10 in the pink gene and 8 and 9 in the gray gene differ by just 3 bp. This suggests that the germline duplication preserved MDS junctions and then two new IESs were inserted into the pink gene after duplication.

Phylogenetic tree reconstruction using just the alternative MDSs permits visualization and inference of the duplication events. Figure 3*C* shows a phylogeny based on the unique regions of two paralogous genes with shared MDSs. The phylogeny suggests that duplication of the alternative MDS region occurred after the divergence of *Paraurostyla*, and that gene B evolved faster than gene A post duplication. There are 11 cases where the phylogenetic analysis suggests that the duplication occurred earlier than would be inferred based on ortholog presence and that one copy was lost in some lineages. The numbers in parenthesis in figure 2*B* show the corrected numbers of inferred origins after examining individual phylogenetic trees.

There are 15 cases where the alternative MDSs show no similarity at the protein level (BLASTP, e-value cutoff 1 e-6), suggesting that they did not arise through duplication. These alternative MDSs could be derived from MIC-limited mobile elements, although their sequences do not correspond to any known transposons in *Oxytricha*. The lower GC content of some of these segments suggests that they could even be derived from retention of MIC-limited noncoding sequences in the MAC (as demonstrated between strains in Möllenbeck et al. 2006, and experimentally in Fang et al. 2012). For 9 of the 15 cases, no stichotrich species contains just one ortholog (precluding our ability to distinguish ancestral from novel genes); however, we could unambiguously assign the novel gene in the other 6 examples (i.e., gene B in fig. 2*A*). Among these, the GC content of the alternative regions in five genes (0.261, 0.305, 0.306, 0.310, 0.310) falls below the lower quartile among all genes in the MAC genome (0.313), suggesting that they may have been acquired from MIC-limited noncoding sequences, which typically have a lower GC content (average 0.284) than the MAC genome.

### Evolution of Alternative and Constitutive MDSs

**Fig. 4.—:**
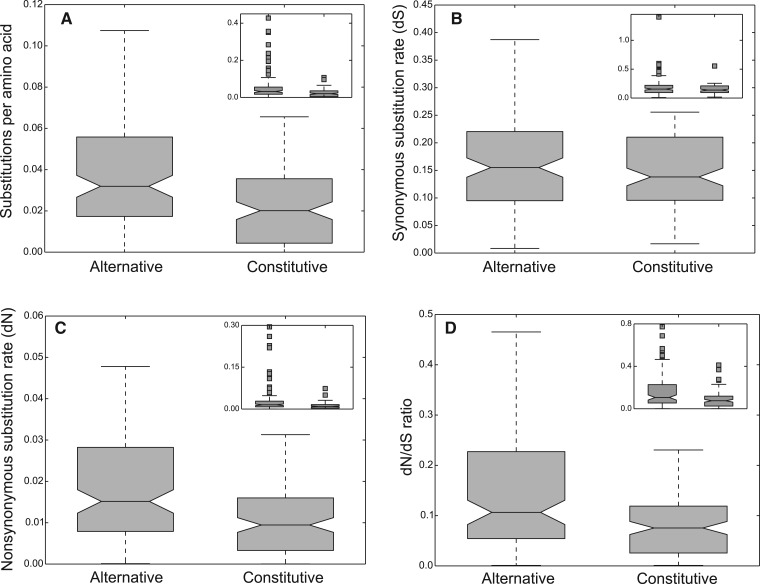
Substitution rates for alternative versus shared gene segments. (*A*) Amino acid substitution rates of alternative versus constitutive MDSs. (*B*) Synonymous substitution rates (d*S*) of alternative versus constitutive MDSs. (*C*) Nonsynonymous substitution rates (d*N*) of alternative versus constitutive MDSs. (*D*) d*N*/d*S* values of alternative versus constitutive MDSs.

We compared the substitution rates between alternative and constitutive MDSs by using amino acid divergence and the ratio of nonsynonymous to synonymous substitution rates (d*N*/d*S*). Because the divergence levels among the ciliate species are so high that the rate of synonymous substitutions per synonymous site (d*S*) is highly saturated, we used the comparison between two *O. trifallax* laboratory strains, JRB310 and JRB510, to infer the d*N*/d*S* ratio. We sequenced and assembled the macronuclear genome of the *O. trifallax* strain JRB510 and compared it with the MAC genome of strain JRB310 reported by Swart et al. (2013). The distance between these strains is suitable for calculating d*N*/d*S* ratios (median dN: 0.0097; median dS: 0.15; median d*N*/d*S*: 0.066). The d*N*/d*S* ratios between JRB310 and JRB510 orthologs only represent evolutionary rates after divergence of the two strains, but not immediately after the formation of new genes. We find that alternative MDSs evolve faster than shared MDSs, with higher amino acid substitution rates (fig. 4*A*, Wilcoxon signed-rank test, *P* = 6.21e-09). There is no significant difference between synonymous substitution rates (fig. 4*B*, *P* = 0.173), but the nonsynonymous substitution rates of alternative MDSs are significantly higher (fig. 4*C*, *P* = 3.3e-6), as well as the d*N*/d*S* ratios (fig. 4*D*, *P* = 3.89e-8). This faster substitution rate is consistent with either stronger functional constraints on the shared regions or, conversely, either weaker selective constraints on the alternative MDSs or greater functional divergence. Shared, constitutive MDSs are intrinsically more constrained because they are translated in more than one gene product, whereas alternative MDSs should have more opportunity to diverge.

### Potential Functional Divergence of Genes with Shared MDSs

Newly created genes sometimes undergo functional divergence (neofunctionalization or subfunctionalization) to acquire different cellular roles, especially genes that arise through duplication (Zhang 2003; Conant and Wolfe 2008). Similarly, functional divergence could be possible for genes with alternative MDSs that arise through duplication or other mechanisms. We investigated whether the new genes that emerged from alternative MDS processing have evolved either different domain organization or expression patterns. Protein domain analysis did not identify any novel combinations of protein domains in our data set of 69 cases. Instead, the unique MDSs for each group either do not contain any recognizable protein domains or encode the same protein domains.

**Fig. 5.—:**
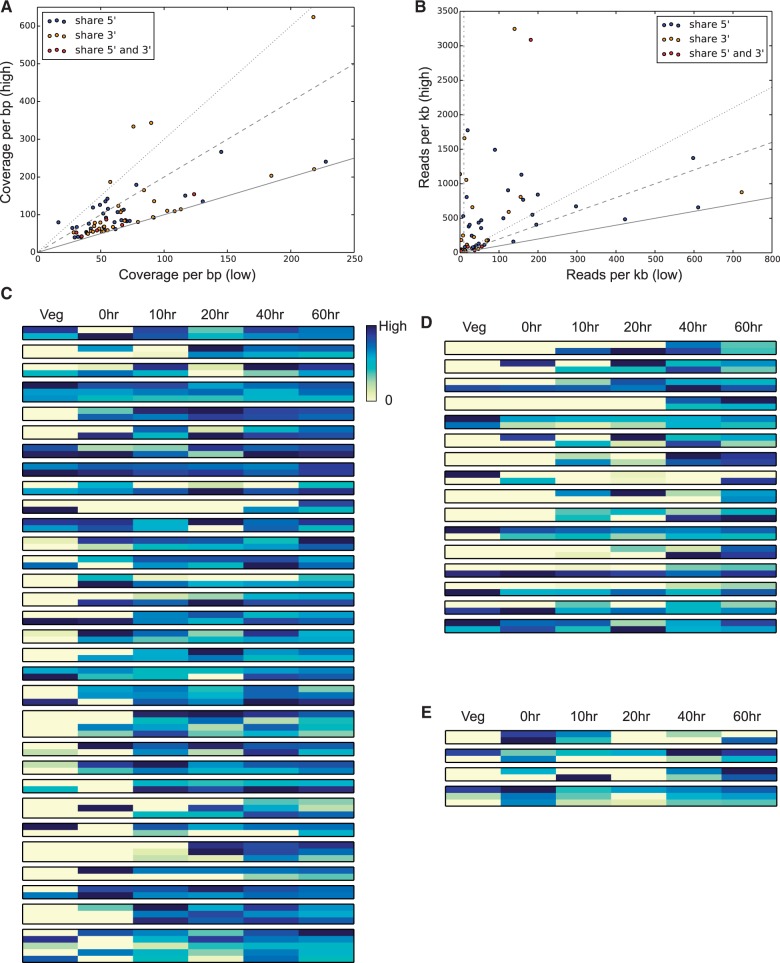
Divergent expression profiles of genes that share precursor segments. (*A*) DNA copy number of genes that share MDSs. For each group of genes that share MDSs, the lowest copy number is plotted on the *x*-axis and the highest copy number on the *y*-axis. The solid, dashed, and dotted lines represent *y* = *x*, *y* = 2*x*, and *y* = 3*x*, respectively. (*B*) Total expression level of genes that share MDSs across a developmental time course. Gene expression levels are represented by a number of normalized RNA-seq counts per kb. The three lines *y* = *x*, *y* = 2*x*, and *y* = 3*x* are as in Panel A. The dashed dotted vertical line represent the cutoff of total expression level of ten normalized RNA-seq reads per kb. (*C*) Gene expression profiles of 31 groups of genes that share 5′ regions. The developmental time course includes six time points: Vegetative, asexually growing stage (Veg) and 0, 10, 20, 40, 60 h post mixing of compatible mating types (strains JRB310 and JRB510) to initiate conjugation and macronuclear development. (*D*) Gene expression profiles of 16 groups of genes that share 3′ regions. (*E*) Gene expression profiles of four groups of genes that share both 5′ and 3′ regions.

Although the DNA copy number for genes with shared MDSs is usually similar to each other (only four show a difference above 3-fold; fig. 5*A*), their overall RNA expression levels differ greatly across all time points during macronuclear development (Swart et al. 2013) (only nine show a difference below 2-fold; fig. 5*B*), suggesting the possibility of distinct or specialized roles. We also compared the expression profiles for genes with shared MDSs by assessing whether their gene expression levels peak at the same time point. We excluded genes that have total normalized expression levels below ten (i.e., ten normalized RNA-seq reads per kb, represented by the dashed dotted vertical line in fig. 5*B*), because low expression may affect the accuracy of the peak analysis. This filter excluded 1 out of 32 cases of genes with shared 5′ DNA regions, 15 out of 31 groups of genes with shared 3′ regions (a higher percentage because RNA-seq is biased toward the 3′ end of a transcript due to poly(A) enrichment during Oligo(dT) priming, but only RNA-seq reads mapping to 5′ ends were scored for these genes), and 2 out of 6 cases of genes that share both 5′ and 3′ regions. For these excluded genes, we verified that their expression is higher than ten normalized RNA-seq reads per kb at other nondevelopmental time points, to exclude the possibility that they are nonfunctional pseudogenes. Among the remaining 31 cases with shared 5′ regions, only 9 show expression peaks at the same time point, and the other 71% have different peaks of expression, consistent with possible functional divergence of the latter cases. Among the remaining 16 cases with shared 3′ regions, only 3 cases have gene expression peaks at the same time point, also suggesting the opportunity for functional divergence among the other 13 cases (81%). Two of the remaining four groups of genes that share both 5′ and 3′ regions have gene expression peaks at the same time point (50%). Figure 5*C*–*E* show distinct expression profiles of genes with shared 5′ or 3′ regions, or both, and that passed the expression filter, suggesting that some new genes created by alternative DNA processing may have undergone functional divergence.

## Discussion

Here, we used a genome-wide survey in the ciliate *Oxytricha* and comparative genomics to demonstrate that alternative DNA processing is a novel mechanism for the origin of new genes. The strongest piece of evidence lies in the observation that there is not a single case where the orthologs of two *Oxytricha* genes that share DNA segments do not share sequences in other ciliate species. This suggests that the emergence of alternative MDS processing is tightly linked to the creation of new genes. All cases of alternative MDS processing in *Oxytricha* have been validated by carefully examining the precise order of DNA segments in the micronuclear genome and mapping micronuclear genomic sequencing reads to assess the copy number of shared MDSs. Because, in *Oxytricha*, the shared, constitutive MDSs are, by definition, always single copy in the germline (and present more than once in the soma), we infer that the orthologs that share sequences in other ciliate species most likely arise from alternative MDS processing. A preliminary micronuclear genome assembly of *Tetmemena* sp. (data not shown) confirms this hypothesis. Sequencing and assembly of the micronuclear genomes of more of these ciliate species will provide future insights into the structural arrangement of constitutive and alternative MDSs in the germline and the mechanisms that drive alternative DNA processing.

Alternative DNA processing in ciliates with intragenic DNA rearrangements is similar, in many respects, to alternative mRNA splicing. Alternatively spliced exons can arise through exon shuffling, mainly via exon duplication, and exonization of noncoding (intronic) sequences (Keren et al. 2010). Likewise, we show here that alternative MDSs appear to arise mainly by MDS duplication and some possibly originate from germline-limited noncoding sequences. An analysis of human, fly, and worm genomes revealed the existence of tandemly duplicated exons in ∼10% of all genes, among which 60% show mutually exclusive alternative splicing of the duplicated exons (Letunic et al. 2002). Most cases that we describe of alternative MDS processing in ciliates are analogous to the mutually exclusive alternative splicing of tandemly duplicated exons. Alternative assembly of tandemly duplicated exons or MDSs provides opportunities to produce modified or divergent protein functions. Both alternative mRNA splicing and alternative MDS processing influence how genes evolve. Alternative exon sequences typically evolve faster than constitutive exons (Chen et al. 2006; Ermakova et al. 2006). Similarly, we find that alternative MDSs evolve faster than shared MDSs. This difference in evolutionary constraints and rates of substitution may contribute to protein diversity in ciliate evolution and their rich gene repertoire. Gene family expansion through MDS duplication and alternative assembly could also help explain the higher number of genes encoded in ciliate genomes than that of most protists and even some metazoans.

Newly created alternative MDSs, that arise from either MDS duplication or incorporation of noncoding sequences, might initially be recognized erroneously by the long template RNAs (Nowacki et al. 2008) that derive from the ancestral genes with the constitutive MDSs. This would allow the new gene arrangements to propagate in the new MAC. The polytene chromosome stage during macronuclear development (Spear and Lauth 1976) allows single-copy, constitutive MDSs to be joined to different groups of alternative MDSs. The new nanochromosomes that incorporate alternative MDSs will then produce their own RNA templates for their maintenance in future generations, consistent with an epigenetic model of RNA-guided genome remodeling (Nowacki et al. 2008).

This study provides the first genome-wide evolutionary analysis of the creation of novel genes through alternative DNA processing and demonstrates the impact of programmed genome rearrangement on new gene origination. Preliminary expression analysis suggests the possibility that some of these genes may have functionally diverged as well, which future experiments can test using the growing arsenal of tools available to *Oxytricha*, such as knockdown of individual genes, as in Fang et al. (2012).

## Supplementary Material

Supplementary table S1 is available *Genome Biology and Evolution* online (http://www.gbe.oxfordjournals.org/).

Supplementary Data
