# Supplementary material for: Combinatorial DNA Rearrangement Facilitates the Origin of New Genes in Ciliates
Source: Genome Biol Evol. 2015 Sep 2;7(10):2859–70. doi: 10.1093/gbe/evv172 (PMC4684698; doi:10.1093/gbe/evv172)
Supplement: Supplementary Data [file supp_7_10_2859__index.html]

Combinatorial DNA Rearrangement Facilitates the Origin of New Genes in Ciliates — Supplementary Data 

# Combinatorial DNA Rearrangement Facilitates the Origin of New Genes in Ciliates

## Supplementary Data

files

- Supplementary Data - txt file
